# Supplementary material for: Global variation in plant-beneficial bacteria in soil under pesticide stress
Source: Nat Commun. 2025 Nov 27;16:10685. doi: 10.1038/s41467-025-65719-7 (PMC12661013; doi:10.1038/s41467-025-65719-7)
Supplement: Supplementary file 1 — Supplementary Information [file 41467_2025_65719_MOESM1_ESM.pdf]

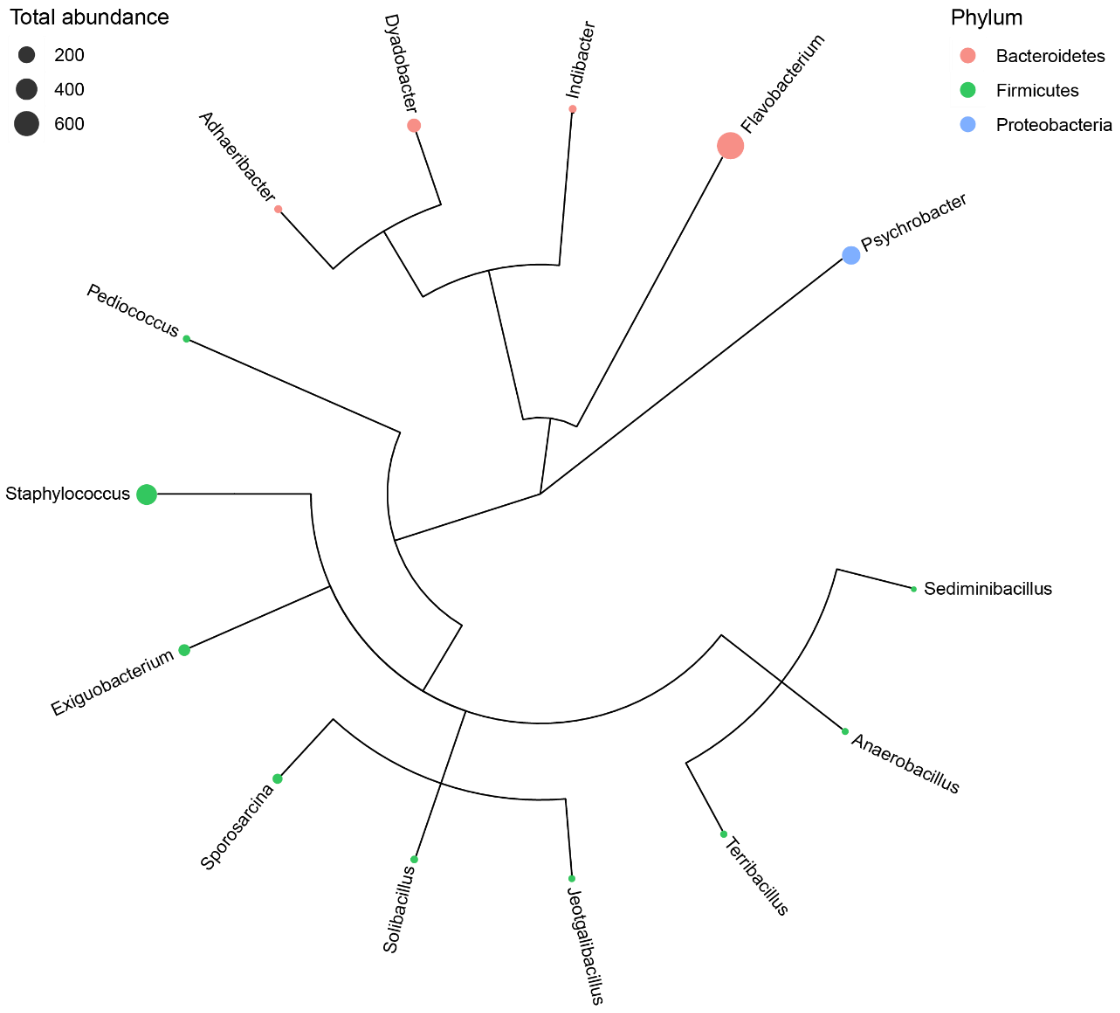

Fig. S1 Taxonomic tree of significantly enriched plant-beneficial bacteria (PBB). Source data are provided as a Source Data file.

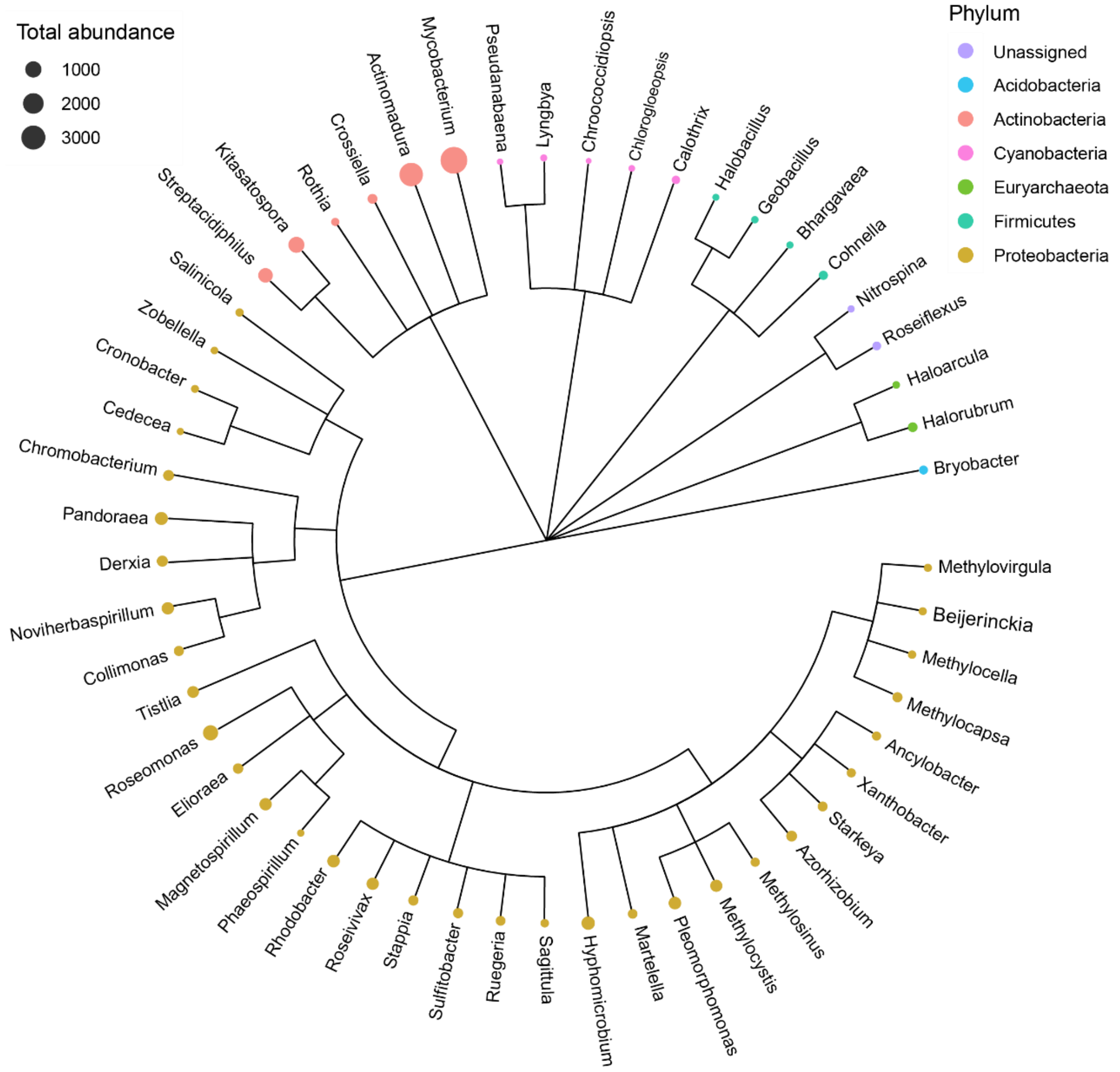

Fig. S2 Taxonomic tree of significantly decreased plant-beneficial bacteria (PBB). Source data are provided as a Source Data file.

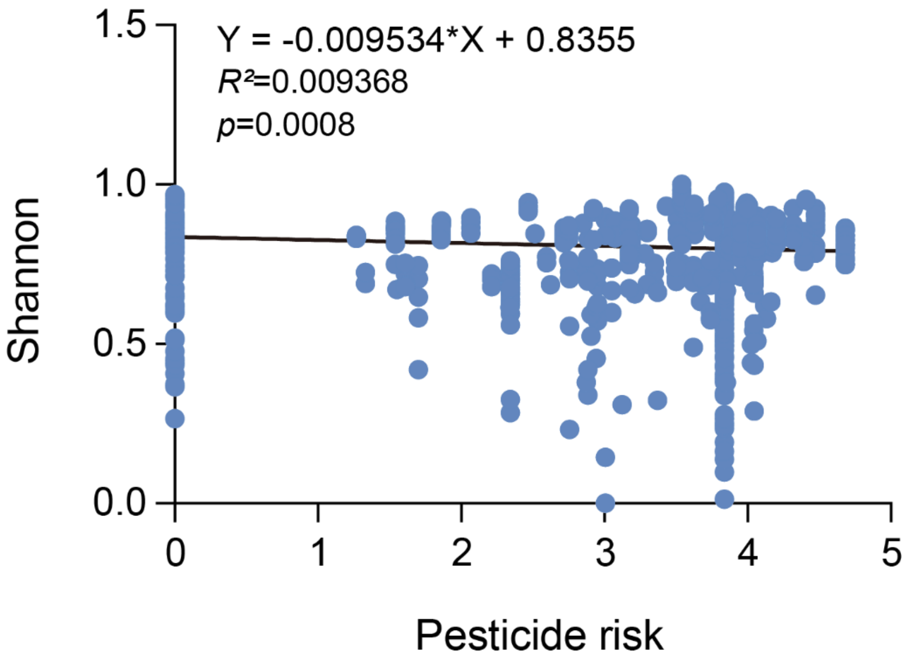

Fig. S3 Univariate linear regression analysis of alpha diversity (Shannon index) of plant-beneficial bacteria community in relation to pesticide risk (n=1191). Source data are provided as a Source Data file.

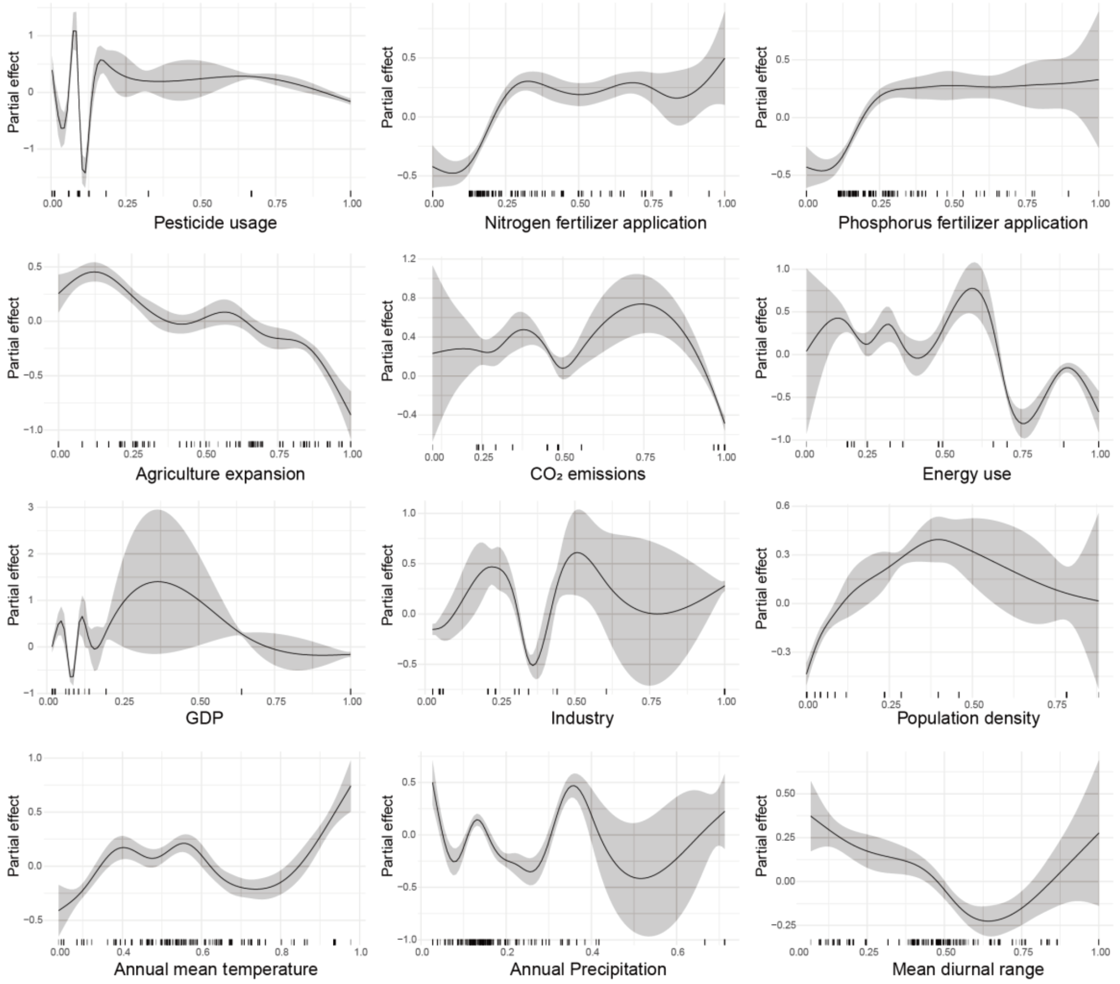

Fig. S4 Nonlinear effects of anthropogenic and environmental predictors on PBB diversity as revealed by generalized additive models (GAMs). Source data are provided as a Source Data file.
