# Supplementary material for: Global variation in plant-beneficial bacteria in soil under pesticide stress
Source: Nat Commun. 2025 Nov 27;16:10685. doi: 10.1038/s41467-025-65719-7 (PMC12661013; doi:10.1038/s41467-025-65719-7)
Supplement: Supplementary file 2 — Description of Additional Supplementary Information [file 41467_2025_65719_MOESM2_ESM.pdf]

Description of Additional Supplementary Files

File Name: Supplementary Data 1
Description: Metadata of 1919 soil metagenomic samples from 88 independent experiments across 237 global sites. Each entry includes BioProject ID, ENA Run ID, habitat type, collection date, geographic coordinates (latitude, longitude), soil type, pesticide risk index (numeric value), pesticide risk class (low, medium, high), country, continent, data source, and DOI. These metadata were used for compiling the global dataset analyzed in this study (n = 1919).

File Name: Supplementary Data 2
Description: Functional pathways of plant-beneficial bacteria (PBB) significantly enriched or reduced with increasing pesticide risk. The dataset lists 18 pathways showing significant changes (Kruskal–Wallis test). "PBB-up" denotes PBB pathways that significantly increase with pesticide risk, while "PBB-down" denotes those that significantly decrease.

File Name: Supplementary Data 3
Description: KEGG-based manual reconstruction of 22 biosynthetic pathways (625 KO terms) associated with growth factor biosynthesis. The reconstructed pathways were grouped into six major biosynthetic categories.

File Name: Supplementary Data 4
Description: Genus-level database of potential plant-beneficial bacteria (PBB) constructed from 1919 samples. The database includes Guild2 classification (beneficial trait categories) and Guild3 classification (beneficial functional categories).

File Name: Supplementary Data 5
Description: Statistical results of spatial analyses assessing the potential confounding effects of spatial autocorrelation on PBB community composition. The dataset summarizes Mantel tests, partial Mantel tests, and distance-based redundancy analysis (db-RDA), including test description, test statistics, p-values, and biological interpretation. Unless otherwise stated, all statistical tests were performed as two-sided.

File Name: Supplementary Data 6
Description: Comparative analysis of functional profiles across pesticide risk levels. The table reports the relative abundance of each COG (Clusters of Orthologous Groups) ID across low-, medium-, and high-risk samples, along with the results of Kruskal–Wallis tests used to identify significantly different COG functions. False discovery rate (FDR) correction was applied to control for multiple testing. KEGGbased functional annotations were included to compare enriched pathways and assess their functional consistency.
