# Supplementary material for: Global variation in plant-beneficial bacteria in soil under pesticide stress
Source: Nat Commun. 2025 Nov 27;16:10685. doi: 10.1038/s41467-025-65719-7 (PMC12661013; doi:10.1038/s41467-025-65719-7)
Supplement: Supplementary file 4 — Reporting Summary [file 41467_2025_65719_MOESM4_ESM.pdf]

nature portfolio

Corresponding author(s): Haifeng Qian
Last updated by author(s): Oct 7, 2025

Reporting Summary

Nature Portfolio wishes to improve the reproducibility of the work that we publish. This form provides structure for consistency and transparency in reporting. For further information on Nature Portfolio policies, see our Editorial Policies and the Editorial Policy Checklist.

Statistics

For all statistical analyses, confirm that the following items are present in the figure legend, table legend, main text, or Methods section.

n/a	Confirmed	
☐	☒	The exact sample size (*n*) for each experimental group/condition, given as a discrete number and unit of measurement
☒	☐	A statement on whether measurements were taken from distinct samples or whether the same sample was measured repeatedly
☐	☒	The statistical test(s) used AND whether they are one- or two-sided<br>*Only common tests should be described solely by name; describe more complex techniques in the Methods section.*
☒	☐	A description of all covariates tested
☐	☒	A description of any assumptions or corrections, such as tests of normality and adjustment for multiple comparisons
☐	☒	A full description of the statistical parameters including central tendency (e.g. means) or other basic estimates (e.g. regression coefficient) AND variation (e.g. standard deviation) or associated estimates of uncertainty (e.g. confidence intervals)
☐	☒	For null hypothesis testing, the test statistic (e.g. *F*, *t*, *r*) with confidence intervals, effect sizes, degrees of freedom and *P* value noted<br>*Give P values as exact values whenever suitable.*
☒	☐	For Bayesian analysis, information on the choice of priors and Markov chain Monte Carlo settings
☒	☐	For hierarchical and complex designs, identification of the appropriate level for tests and full reporting of outcomes
☐	☒	Estimates of effect sizes (e.g. Cohen's *d*, Pearson's *r*), indicating how they were calculated

Our web collection on statistics for biologists contains articles on many of the points above.

Software and code

Policy information about availability of computer code

Data collection	All the raw data in this study were collected in public repositories including the European Nucleotide Archive (https://ebi.ac.uk/ena/), the National Center for Biotechnology Information (NCBI; https://ncbi.nlm.nih.gov/sra/), and the Metagenome Rapid Annotation via Subsystem Technology (https://www.mg-rast.org/).. IBM® Aspera Connect (v4.1.1) was used for downloading all these data.
Data analysis	Raw sequencing reads were first processed for quality control by using Trimmomatic (v2.39)27 to remove adapter and primer sequences, discard reads shorter than 50 bp, and trim low-quality bases (quality score < 20). Cleaned reads were then evaluated using FastQC (v0.11.5; https://github.com/s-andrews/FastQC) to ensure that the data quality fulfilled the requirements for subsequent assembly. Quality-controlled reads were assembled de novo into contigs by using MEGAHIT28 (v1.2.8, https://github.com/voutcn/megahit), using appropriate k-mer settings to balance accuracy and contig length. Assembly quality was subsequently assessed to ensure the completeness and accuracy of the assembled contigs. The assembled contigs were grouped into draft genomes by using Metabat2, which clusters contigs based on their GC content and read coverage to separate the microbial genomes. The bin quality was further evaluated using CheckM (v1.1.6; https://github.com/Ecogenomics/CheckM/wiki) to ensure that completeness and contamination levels were within acceptable thresholds, and high-quality bins were selected for further annotation. Taxonomic classification of the binned genomes was performed using Kraken2 (v2.1.2)29, that assigns each genome to an appropriate taxonomic rank (e.g., phylum, genus, or species). This step provided insights into the microbial community structure within the soil sample.Functional annotation of the contigs was performed by mapping to the Kyoto Encyclopedia of Genes and Genomes (KEGG) database. This process identified key functional genes and metabolic pathways, allowing the analysis of the metabolic capabilities and ecological roles of the microbial community within the soil environment. We annotated the data by using a comprehensive database of genes involved in the cycling of carbon, nitrogen, phosphorus, and sulfur30. We used Diamond (v2.0.14)31 with the parameters e-value = 0.001, coverage = 60%, and identity = 70% to align the contigs with the elemental cycling database to annotate their potential functions. Based on the KEGG database, we manually reconstructed 22 biosynthetic pathways comprising 625 KO terms (Supplementary data 3). In addition to the KEGG-based functional annotation, we have conducted parallel functional assignments using the

COG database (COG2024, NCBI). Protein-coding sequences (CDSs) predicted from the assembled contigs were aligned against the COG2024 reference database (COGorg24.faa) using DIAMOND BLASTP (v2.1.3) with default parameters. The best hits were assigned COG identifiers based on their alignment with cog-24. cog.csv file. Functional categories and descriptions were annotated using cog-24. def.tab and cog-24. We calculated the relative abundance of each COG ID in each sample and also performed Kruskal–Wallis tests in order to detect significantly different COG functions across the pesticide risk levels. Significantly enriched pathways were compared using KEGG-based annotations to evaluate their functional consistency (Supplementary data 6 for detailed results).While we did not directly benchmark our workflow against other metagenomic analysis platforms, such as MG-RAST or QIIME2, we used widely adopted and validated tools (e.g., Kraken2 for taxonomic assignment) that are commonly used in large-scale soil microbiome studies. Our workflow was specifically designed to ensure high-throughput compatibility, scalability, and consistency across 1,919 metagenomic datasets. Reproducibility was prioritized by publicly sharing all scripts, parameters, and documentation on GitHub.The R and Python codes used in this study are available online at https://github.com/QDY742/Global-variation-of-plant-beneficial-bacteria-under-pesticide-stress.

For manuscripts utilizing custom algorithms or software that are central to the research but not yet described in published literature, software must be made available to editors and reviewers. We strongly encourage code deposition in a community repository (e.g. GitHub). See the Nature Portfolio guidelines for submitting code & software for further information.

Data

Policy information about availability of data

All manuscripts must include a data availability statement. This statement should provide the following information, where applicable:

- Accession codes, unique identifiers, or web links for publicly available datasets
- A description of any restrictions on data availability
- For clinical datasets or third party data, please ensure that the statement adheres to our policy

All metagenomic datasets analyzed in this study (n = 1,919) were retrieved from public repositories including National Center for Biotechnology Information (NCBI, https://ncbi.nlm.nih.gov/sra/), European Nucleotide Archive (https://ebi.ac.uk/ena/), and Metagenome Rapid Annotation via Subsystem Technology (https://www.mg-rast.org/). Detailed metadata, including BioProject IDs, ENA Run IDs, collection sites, and environmental parameters, are provided in Supplementary Data 1. No new metagenomic data were generated in this study; therefore, no MG-RAST Project ID is applicable. Supplementary Data containing the critical supplementary information in this study are publicly available online (https://doi.org/10.6084/m9.figshare.30276679). The raw data underlying figures are provided as Source data which can be obtained in public repository (https://doi.org/10.6084/m9.figshare.30276595). Source data are provided with this paper.

Research involving human participants, their data, or biological material

Policy information about studies with human participants or human data. See also policy information about sex, gender (identity/presentation), and sexual orientation and race, ethnicity and racism.

Reporting on sex and gender	No human-related data were used in this research
Reporting on race, ethnicity, or other socially relevant groupings	see above
Population characteristics	see above
Recruitment	see above
Ethics oversight	see above

Note that full information on the approval of the study protocol must also be provided in the manuscript.

Field-specific reporting

Please select the one below that is the best fit for your research. If you are not sure, read the appropriate sections before making your selection.

☐ Life sciences ☐ Behavioural & social sciences ☒ Ecological, evolutionary & environmental sciences

For a reference copy of the document with all sections, see nature.com/documents/nr-reporting-summary-flat.pdf

Ecological, evolutionary & environmental sciences study design

All studies must disclose on these points even when the disclosure is negative.

Study description	This study investigates the impact of pesticide pollution on plant-beneficial bacteria (PBB) in soil, which are crucial for crop production and soil health. Utilizing metagenomic data from 1,919 soil samples, researchers identified 364 PBB species and analyzed their diversity and functional roles.
Research sample	The keywords “soil AND metagenome,” “soil AND microbiome,” “farmland AND metagenome,” “soil AND metagenomic,” “land AND metagenome OR metagenomic,” and “soil metagenome OR metagenomic” were searched to obtain the required soil metagenomic data. The databases used included the European Nucleotide Archive (https://ebi.ac.uk/ena/), the National Center for Biotechnology Information (NCBI; https://ncbi.nlm.nih.gov/sra/), and the Metagenome Rapid Annotation via Subsystem Technology (https://www.mg-rast.org/).<br>Metagenomic data selection was performed based on the following requirements: (1) samples with complete information (including

	habitat type, country, latitude and longitude, data source, and biological project number), (2) samples with only pesticide-contaminated exogenous contaminants in the experimental set, and (3) samples that did not contain plant holobionts. The aim of these selection requirements was to reduce uncertainty in subsequent analyses by controlling for variables such as sequence quality, sequencing method, experimental protocol, and platform. In total, 1,919 samples from 237 global sites were obtained (Supplementary data 1). All raw data are publicly accessible, with file sizes exceeding 2 gigabytes.
Sampling strategy	Metagenomic data were selected from public databases according to strict criteria: (1) samples with complete metadata (habitat type, country, latitude/longitude, data source, project number), (2) exclusion of those with pesticide-unrelated exogenous contaminants, and (3) exclusion of samples closely linked to the plant holobiont (e.g., root, stem, leaf endophytes). These requirements minimized uncertainty by controlling for sequencing quality, methodology, and platform variability. A total of 1,919 soil samples from 237 global sites were included (Supplementary Data 1). No formal sample size calculation was performed; the large dataset and broad geographic coverage were considered sufficient to ensure statistical robustness.
Data collection	The 1919 metagenomic samples were downloaded by QDY, WY, XNH and CBF from European Nucleotide Archive.All the downloaded were used the IBM Aspera Data Transfers service.
Timing and spatial scale	The 1919 metagenomic samples were downloaded in April 2023. The spatial scale from which the data are taken were mentioned in the Supplementary Table S1, because it is impossible for us to provided all the spatial scale for all the samples here.
Data exclusions	There was no data exclusion in this analysis.
Reproducibility	All the scripts and codes for gene annotation, statistical analysis and visualization used in this study are available online at https://github.com/QDY742/Global-variation-of-plant-beneficial-bacteria-under-pesticide-stress.The use of the data in supplementary data 1 enabled the replication of our study.
Randomization	Not relevant to this study, as all the data we used were public.
Blinding	Blinding is not used in this study, because all of our data were public and is not influenced by the observer.

Did the study involve field work? ☐ Yes ☒ No

Reporting for specific materials, systems and methods

We require information from authors about some types of materials, experimental systems and methods used in many studies. Here, indicate whether each material, system or method listed is relevant to your study. If you are not sure if a list item applies to your research, read the appropriate section before selecting a response.

Materials & experimental systems

n/a	Involved in the study	
☒	☐	Antibodies
☒	☐	Eukaryotic cell lines
☒	☐	Palaeontology and archaeology
☒	☐	Animals and other organisms
☒	☐	Clinical data
☒	☐	Dual use research of concern
☒	☐	Plants

Methods

n/a	Involved in the study	
☒	☐	ChIP-seq
☒	☐	Flow cytometry
☒	☐	MRI-based neuroimaging

Plants

Seed stocks	No plants were used in this study for experiments
Novel plant genotypes	No plants were used in this study for experiments
Authentication	No plants were used in this study for experiments
